# A novel missense in GLI3 possibly affecting one of the zinc finger domains may lead to postaxial synpolydactyly: case report

**DOI:** 10.1186/s12881-019-0889-5

**Published:** 2019-11-09

**Authors:** Qianqian Zou, Zhigang Tian, Jie Zheng, Xiufang Zhi, Xiaojie Du, Jianbo Shu, Chunquan Cai

**Affiliations:** 1 Graduate College of Tianjin Medical University, Tianjin, China; 2 Department of Orthopaedics, Tianjin Children’s Hospital, Tianjin, China; 3 Institute of Paediatrics, Tianjin Children’s Hospital, No. 238 Longyan Road, Beichen District, Tianjin, China; 4 Department of Neurosurgery, Tianjin Children’s Hospital, No. 238 Longyan Road, Beichen District, Tianjin, China

**Keywords:** Synpolydactyly, *GLI3* gene, Gene mutation

## Abstract

**Background:**

Polydactyly is one of the most common congenital hand/foot malformations in humans. Mutations in *GLI3* have been reported to cause syndromic and non-syndromic forms of preaxial and postaxial polydactylies.

**Case presentation:**

The patient was a 2-year-old boy who underwent surgery in our hospital. The right hand and left foot of the patient were labelled as postaxial polydactyly type B, and there was cutaneous webbing between the 3rd and 4th fingers of the left hand. We identified a novel c. 1622C > T variant in *GLI3* leading to an isolated postaxial synpolydactyly.

**Conclusions:**

The patient carries a novel autosomal dominant heterozygous missense mutation. This mutation c.1622C > T;p.(Thr541Met) in the *GLI3* gene may affect the normal function of the zinc finger domain (ZFD) in a different way. However, it seems that more research is needed to determine the exact effects of this mutation.

## Background

Polydactyly is one of the most common hand-foot malformations in humans. Neonatal morbidity in the world is approximately 0.3/1000–3.6/1000 and is approximately twice as high for males as for females [1]. This malformation can occur alone (non-syndromic type) and can also be combined with a variety of other symptoms (syndromic type) [2]. Genotype-phenotype correlation studies suggest that truncation mutations upstream of or within the zinc finger domain (ZFD) usually result in Greig cephalopolysyndactyly syndrome (GCPS). Protein truncation in the middle portion of the *GLI3* protein is associated with Pallister–Hall syndrome (PHS). Mutations that cause truncations in the C-terminal part of the *GLI3* protein result in a variable phenotype of GCPS, postaxial polydactyly types A or B (PAP A/B) or preaxial polydactyly type IV (PPD IV) [3]. In this study, we report a boy with isolated postaxial synpolydactyly who carries a novel autosomal dominant heterozygous missense mutation near the ZFD of *GLI3*.

## Case presentation

**Fig. 1:**
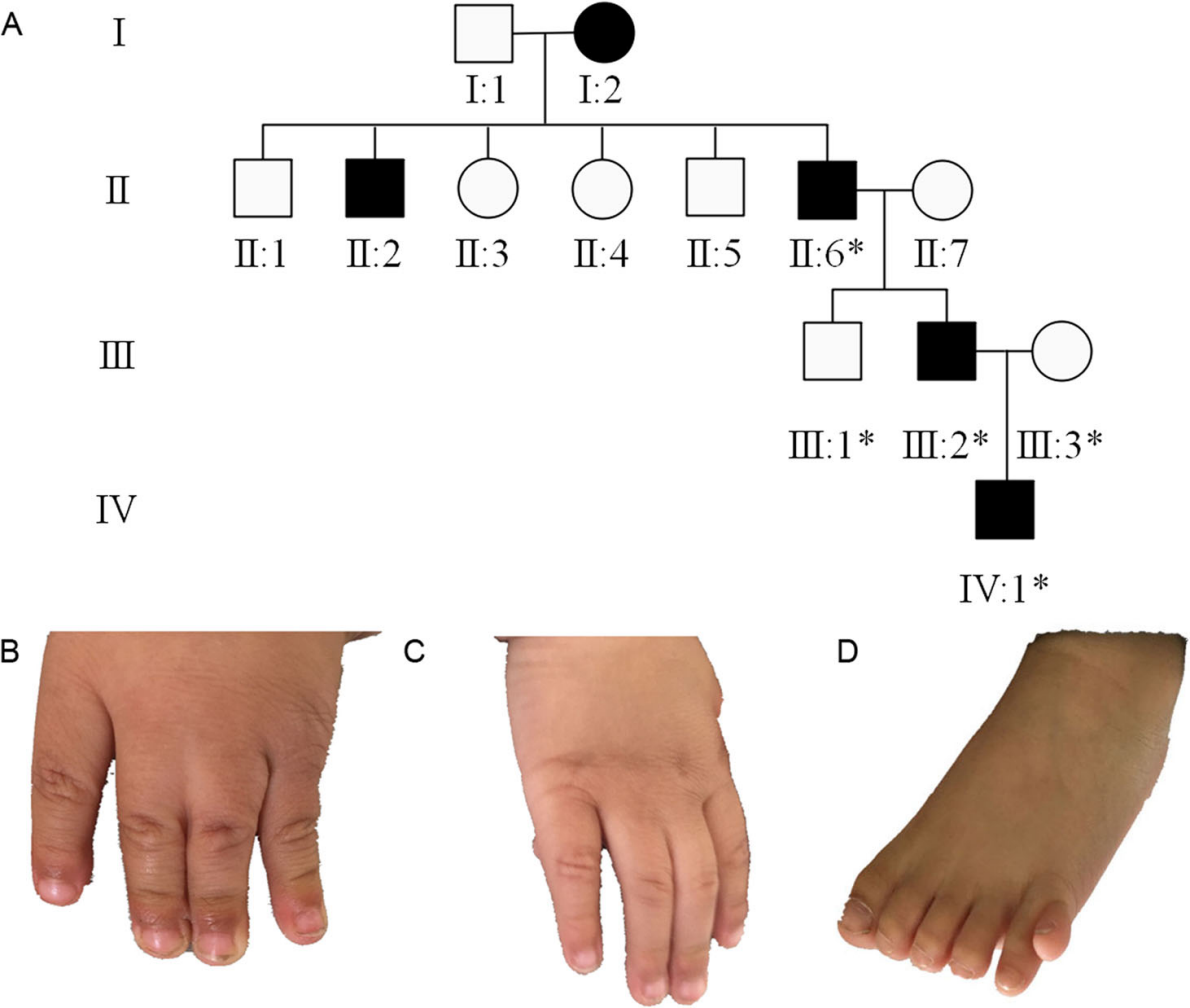
The clinical phenotype. **a**. Family tree of the studied individuals (the individuals included in this study are specified by asterisks). Both hands of the proband’s grandfather were classified as post-axial polydactyly type B (individual II:6). The father’s left hand was classified as post-axial polydactyly type B (individual III:2). The patient’s right hand was classified as post-axial polydactyly, and his left hand had cutaneous webbing between the 3rd and 4th fingers; his left foot had a well-formed digit on the fibular aspect (individual IV:1) (**b**, **c** and **d**)

**Fig. 2:**
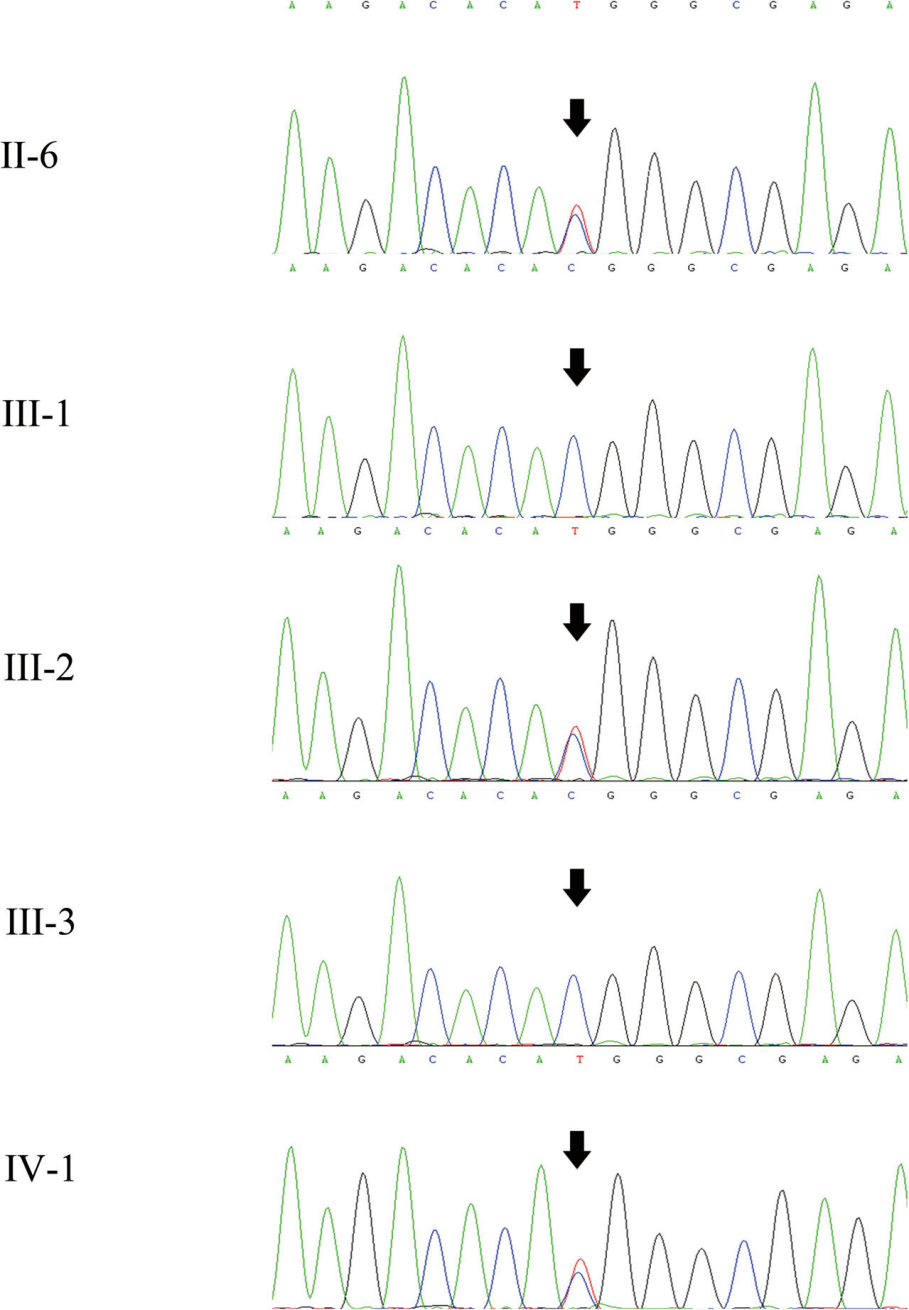
Sequencing data of the *GLI3* gene in the patient and his family. The arrows indicate the position of the c.1622C > T; p.(Thr541Met) substitution

**Fig. 3:**
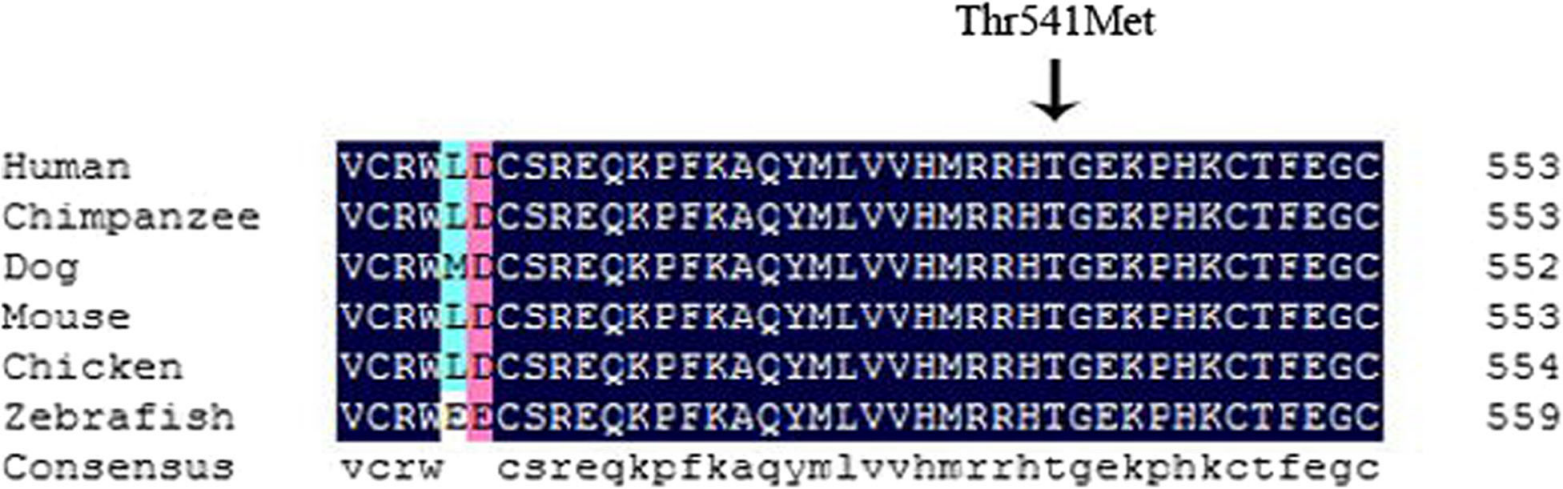
p.Thr541 in GLI3 is highly conserved among at least seven species. The arrows indicate the position of the p.Thr541Met substitution

**Fig. 4:**
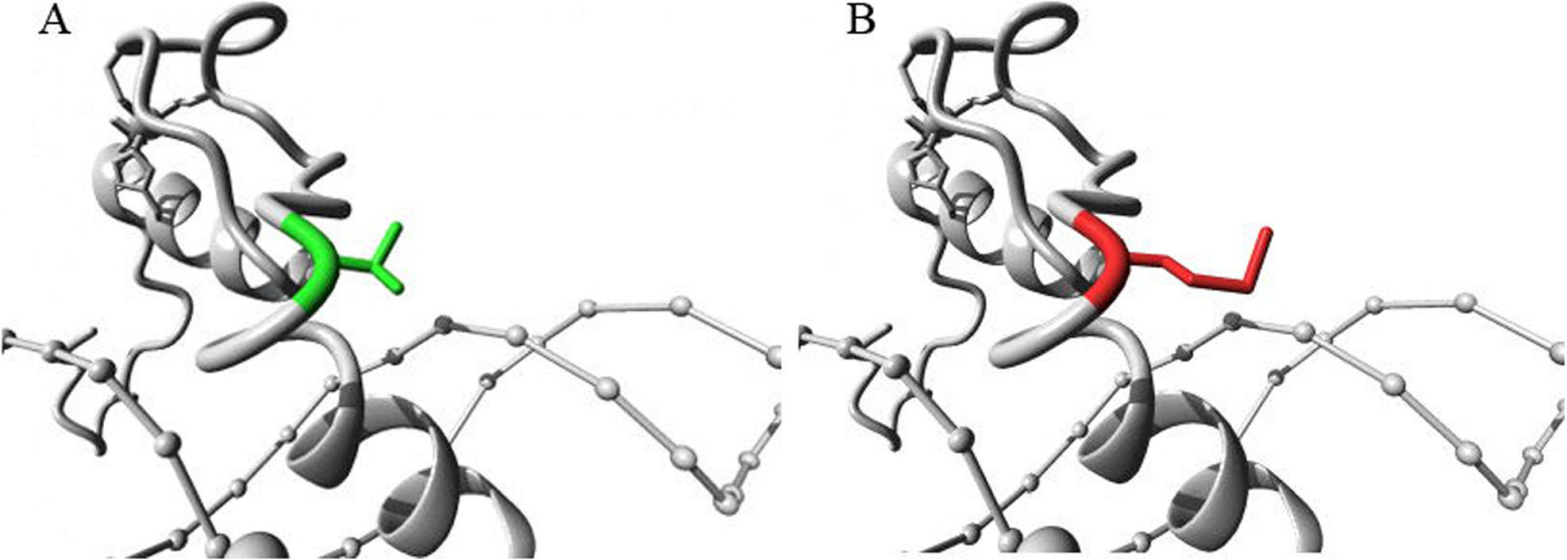
Structural modelling of the predicted wild type (**a**) and mutant p. Thr541Met (**b**) proteins. The mutation is near the second zinc finger

The patient was a 2-year-old boy who underwent surgery in the Department of Orthopaedics, Tianjin Paediatric Hospital. The right hand and left foot of the patient were labelled as PAP B, and there was cutaneous webbing between the 3rd and 4th fingers of the left hand. Phenotypic variability among the other 4 affected related individuals (great-grandmother, granduncle, grandfather, and father) was evident. Their hands were diagnosed as PAP B. The affected individuals did not have craniofacial dysmorphism (Fig. 1). A novel autosomal dominant heterozygous *GLI3* variant, NC_000007.14(NM_000168.5):c.1622C > T; p.(Thr541Met), located in exon 11, was identified by Sanger sequencing of the patient’s gDNA sample. His father and grandfather are heterozygous for the mutation, and his great-grandmother has passed away (Fig. 2; Table 1). This variant was predicted to be most likely damaging, with a score of 1 (sensitivity: 0; specificity: 1) by Polyphen 2 [4]. Moreover, it is forecast to affect protein function, with a score of 0.00 according to SIFT [5]. The *GLI3* variant is highly conserved in the evolution of various species, suggesting the functional importance of this protein (Fig. 3). The simplified model illustrating the mutation in *GLI3* located near the second zinc finger structure in the ZFD was constructed with HOPE (Fig. 4).

**Table 1:** Clinical features of the affected individuals

Affected individual	Sex	Age	Symptoms	Mutation
Great-grandmother (individual I:2)	Female	Deceased	Both hands were classified as post-axial polydactyly type B	–
Granduncle (individual II:2)	Male	56 years	Both hands were classified as post-axial polydactyly type B	–
Grandfather (individual II:6)	Male	54 years	Both hands were classified as post-axial polydactyly type B	c.[1622C > T];p.[(Thr541Met)]
Father (individual III:2)	Male	34 years	Left hand was classified as post-axial polydactyly type B	c.[1622C > T];p.[(Thr541Met)]
Patient (individual IV:1)	Male	2 years	Right hand was classified as post-axial polydactyly, left hand had cutaneous webbing between the 3rd and 4th fingers, left foot had a well-formed digit on the fibular aspect.	c.[1622C > T];p.[(Thr541Met)]

## Discussion and conclusions

Congenital limb abnormalities are the most common birth defects in new-borns. Polydactyly is one of the most common congenital limb malformations [2]. Human embryos start to form at the end of the fourth week of embryonic development. After approximately 4 weeks, the interactions of the genes and various factors play decisive roles in the formation of normal morphology, function and finger number [6]. During the formation of limbs, there are three interacting signalling centres that direct the formation of fingers. Abnormal genes in these signalling centres will result in congenital limb malformations. Recent studies have focused on genes and gene families related to limb development, such as *SHH*, *ZRS* and *GLI3* [7]. Among them, *GLI3* is currently known to be an important signalling molecule that regulates the anteroposterior axis direction [the direction of the first finger (toe) to the fifth finger (toe)] in human embryo development [8].

The *GLI3* gene is located in the 7p14.1 region of the chromosome and consists of 14 exons. The mRNA of *GLI3* is 8.5 kb in length and encodes a polypeptide chain consisting of 1580 amino acids [9]. The protein can be divided into 3 parts that are associated with different genotype-phenotype correlations: the ZFD, the cyclic AMP-binding protein-binding domain (CBPD), and the transactivation domains 1 and 2 (TA1 and 2). The ZFD contains five highly conserved tandem zinc finger structures with specific DNA sequence affinity (2 cysteine and 2 histidine, C2H2). It is a central zinc finger transcription factor in the early development of vertebrate limbs [10]. The mutation is near one of the C2H2s, which contains the 513–540 region in *GLI3*. In some cases, mutations of the ZFD usually result in a reduced expression of *GLI3*, which leads to an expanded expression of the active forms of *GLI3* (*GLI3A*) compared to the repressor forms of *GLI3* (*GLI3R*)*.* These point mutations throughout the *GLI3* gene are well known for causing the GCPS phenotype. Instead, the boy we report carries a novel autosomal dominant heterozygous missense mutation NC_000007.14(NM_000168.5):c.1622C > T; p.(Thr541Met) near the ZFD of *GLI3* associated with isolated postaxial synpolydactyly. Some articles have suggested that the perturbation of the balance of GLI3R and GLI3A is connected to postaxial polydactyly. A mildly abnormal ratio of GLI3R to GLI3A can result in isolated postaxial polydactyly. Therefore, the mutation in our case may affect the normal function of the ZFD in a different manner. Further experiments will be necessary to confirm how this mutation works. We believe that there is practical significance for further studies of synpolydactyly.

## Data Availability

The datasets used and analysed during the current study are available from the corresponding author on reasonable request.

## Abbreviations

C2H2: 2 cysteine and 2 histidine
CBPD: Cyclic AMP-binding protein-binding domain
GCPS: Greig cephalopolysyndactyly syndrome
*GLI3A*: The active forms of *GLI3*
*GLI3R*: The repressor forms of *GLI3*
PAP A/B: Postaxial polydactyly types A or B
PHS: Pallister-Hall syndrome
PPD IV: Preaxial polydactyly type IV
TA1 and 2: Transactivation domain 1 and 2
ZFD: Zinc finger domain
